# Atractylon treatment prevents sleep-disordered breathing-induced cognitive dysfunction by suppression of chronic intermittent hypoxia-induced M1 microglial activation

**DOI:** 10.1042/BSR20192800

**Published:** 2020-06-15

**Authors:** Yan Lin, Xiuxiu Liu, Dan Tan, Zhiyan Jiang

**Affiliations:** Department of Pediatrics, Longhua Hospital, Shanghai University of Traditional Chinese Medicine, 725 South Wan-Ping Road, Shanghai 200065, China

**Keywords:** atractylon, chronic intermittent hypoxia, cognitive dysfunctio, microglial, sleep-disordered breathing

## Abstract

Chronic intermittent hypoxia (CIH) induced by sleep-disordered breathing (SDB) is a key factor involved in cognitive dysfunction (CD). Increasing evidence has shown that atractylon (ATR) has anti-inflammatory effects. However, it remains unclear if ATR has a protective effect against SDB-induced nerve cell injury and CD. So, in the present study, CIH-exposed mice and CIH-induced BV2 cells were used to mimic SDB. The results showed that ATR treatment decreased CIH-induced CD and the expression of inflammatory factors in the hippocampal region by suppression of M1 microglial activation and promotion of M2 microglial activation. Also, ATR treatment promoted sirtuin 3 (SIRT3) expression. Down-regulation of SIRT3 decreased the protective effect of ATR against CIH-induced microglial cell injury. Furthermore, *in vitro* detection found that SIRT3 silencing suppressed ATR-induced M2 microglial activation after exposure to CIH conditions. Taken together, these results indicate that ATR treatment prevents SDB-induced CD by inhibiting CIH-induced M1 microglial activation, which is mediated by SIRT3 activation.

## Introduction

Sleep-disordered breathing (SDB) is a common disease that results in sleep fragmentation and chronic intermittent hypoxia (CIH), approximately 6% of women and 13% of men suffer from the disease [8,16]. Severe SDB impairs quality-of-life by increasing the risks of traffic accidents, cardiovascular disorders, and hypertension [10,12]. Furthermore, SDB has been correlated with the aggressiveness of cancers of the breast [3] and lung [6]. More recently, SDB has been associated with cognitive dysfunction (CD) [5,15]. However, the relative underlying mechanisms remain unclear.

The extracts of *Atractylodes japonica Koidzumi* is used for the treatment of pain, gastritis, and enteritis [21]. The study also found that *Atractylodes rhizomes* have a significant liver-protective effect by suppression of *tert*-butyl hydroperoxide-induced toxicity and DNA damage of hepatocytes [9]. Increasing evidence has also shown that atractylon (ATR), the core component of *A. rhizomes*, has anti-inflammatory effects [4]. Previous studies have found that CD relative to neuroinflammation, when stimulated by external injury or hypoxia, promoted SDB [20,22] and suggested that ATR treatment can prevent SDB-induced CD.

In the present study, we hypothesized that ATR might reduce the inflammatory response. For this purpose, we investigated the protective effects and mechanisms of ATR using CIH-induced mice and CIH-induced BV2 cells.

## Materials and methods

### Animals and ethics statement

BALB/c mice from Shanghai Sippr-Bk Laboratory Animals Co., Ltd. (Shanghai, China) were housed at 24–26 °C under a 12-h light/dark cycle. Animal Ethics Committee of Longhua Hospital (Shanghai, China) approved all animal experiments. The Ethical code was 2018082306A. All animal experiments are done at Longhua Hospital (Shanghai, China). All surgical procedures were performed under anesthesia, and every effort was made to minimize suffering. Mice were anesthetized by intraperitoneal injection of 30 mg/kg sodium pentobarbital.

### CIH exposures

Mice were fed a regular chow diet. The Hycon system (S. Savransky, West Des Moines, IA, U.S.A.) was used to regulate air/N_2_ delivery, which consisted of 90 s of 6.1% O_2_ balance nitrogen alternating with 90 s of 21% O_2_ (room air) for 12 h during the light phase. The O_2_ concentration was adjusted every 12 h (07:00 am to 07:00 pm). Oxygen concentrations were maintained at 21% at nighttime but 6.1% at daytime and lasted for 12 weeks for CIH experiment. Control group was housed in the normal condition (21% O_2_). ATR treatment group received ATR (25 mg/kg) once a week. Each group consisted of six mice.

### Morris water maze test

Learning and memory function was detected as in previous study using Morris water maze (MWM) test [13]. Testing was carried out after 12 weeks of CIH exposure and was continued for 5 days. The first 4 days were used for navigation (reference memory) test. A video tracking system was used to record escape latency of each mouse. On day 5, a spatial probe test was performed for MWM test (Shanghai Mobile Datum Information Technology Co., Shanghai, China).

### Immunofluorescence analysis

Hippocampal tissue was fixed (10% formalin) and embedded in paraffin. To evaluate histopathological changes in the hippocampus, 5 μm-thick sections were stained with antibodies (Abs) against CD11b and ionized calcium-binding adapter molecule 1 (IBA1). The apoptosis was analysed using commercial TUNEL enzyme-linked immunosorbent assay (ELISA) kits (Roche Diagnostics GmbH, Mannheim, Germany). Sections were examined under a fluorescence microscope (Nikon Corporation, Tokyo, Japan).

### BV2 cell culture and activation

BV2 cells (Wuhan Biofavor Biotechnology Service Co., Ltd, Wuhan, China) were maintained in Dulbecco's modified Eagle's medium (DMEM; Invitrogen Corporation, Carlsbad, CA, U.S.A.) supplemented with 10% fetal bovine serum (Invitrogen Corporation, Carlsbad, CA, U.S.A.) in a 5% CO_2_ incubator. Before incubation with or without ATR, the cells were seeded into the wells of a 24-well plate at a density of 1 × 10^5^ cells/ml with serum-free DMEM and cultured overnight. For CIH treatment, the cells were pretreated with or without ATR (25 μg/ml) for 12 h then maintained with 5% CO_2_ under 37 °C (OxyCycler model A42; BioSpherix, Ltd., Parish, NY, U.S.A.); O_2_ levels in incubator were alternated between 1% for 10 min and 21% for 5 min for 18 h. After hypoxic treatment, the medium was replaced and the BV2 cells were returned to normal conditions in an incubator for 60–80 min to induce recovery.

### Small interfering RNA transfection

BV2 cells were transfected with small interfering RNA (siRNA) against sirtuin 3 (siSIRT3; GeneCopoeia, Rockville, MD, U.S.A.) using Lipofectamine 2000 Transfection Reagent (Invitrogen Corporation, Carlsbad, CA, U.S.A.) then cultured for 48 h before the further experiments.

### RNA extraction and quantitative reverse transcription PCR

RNA from hippocampal tissue and BV2 cells was isolated using TRIzol reagent (TaKaRa, Osaka, Japan). The PrimeScript^™^ RT Master Mix kit (TaKaRa) was used for first-strand cDNA synthesis and quantitative reverse transcription PCR (qRT-PCR) was performed with Power SYBR Green PCR Master Mix (Life Technology, U.S.A.) and the following primer sequences: si-SIRT1 antisense: 5′-CAG CAA CCU UCA GCA GUA UUU-3′; control siRNA sense: 5′-AUA CUG CUG AAG GUU GCU GUU-3′. Glyceraldehyde 3-phosphate dehydrogenase (GAPDH) was used as an internal control. The 2^−ΔΔCt^ method was used for gene expression analysis.

### Western blot analysis

Protein extracts from different groups of cells were separated by 10% sodium dodecyl sulfate-polyacrylamide gel electrophoresis before transferred to polyvinylidene fluoride (PVDF) membranes, and then incubated with the corresponding primary Abs at 4°C overnight after blocking with 5% skim milk. The following primary Abs were used: monoclonal Ab against GAPDH (1:1000; Santa Cruz Biotechnology, Santa Cruz, CA, U.S.A.), SIRT3 (1:600; Cell Signaling Technology), nuclear factor (NF)-κB (1:600; Santa Cruz Biotechnology), interferon-γ (IFN-γ; 1:600; Santa Cruz Biotechnology), arginase-1 (Arg1; 1:600; Santa Cruz Biotechnology), inducible nitric oxide synthase (iNOS; 1:600; Santa Cruz Biotechnology), chitinase 3-like 3 (Ym1; 1:600; Santa Cruz Biotechnology), and Iba-1 (1:600; Santa Cruz Biotechnology). The sample were incubated with secondary Abs after incubated with the primary Abs. Subsequently, the membranes were washed and visualized using an electrochemiluminescence system. Protein band densities were analyzed using UN SCAN-IT gel software. β-actin was used as a loading control.

### Flow cytometry

The apoptosis rate of BV2 cells was analyzed using flow cytometry with annexin V-fluorescein isothiocyanate (AV-FITC) and propidium iodide (PI) staining. Cells were washed with cold D-Hank's buffer, and then AV-FITC (10 μl) and PI (10 μl) were added to 100 μl of cell suspension (1×10^6^ cells/ml) and incubated at room temperature for 15 min in the dark condition. Finally, 400 μl of binding buffer was added to each sample for flow cytometry detection.

### Inflammatory cytokines analysis

The expression levels of the inflammatory factors tumor necrosis factor (TNF)-α, interleukin (IL)-6, and IL-1β in brain tissue or cell supernatants were measured using commercially available ELISA kits (Elabscience Biotechnology Co., Ltd, Wuhan, China) according to the manufacturer's instructions.

### Statistical analysis

Statistical comparisons were performed with one-way analysis of variance followed by Bonferroni-corrected pairwise comparisons using GraphPad Prism 5.0 software (GraphPad Software, Inc., La Jolla, CA, U.S.A.). A post-hoc probability (*P*) value of <0.05 was considered significant.

## Results

### ATR treatment decreased CIH-induced CD and expression of inflammatory factors in hippocampal tissue

**Figure 1:**
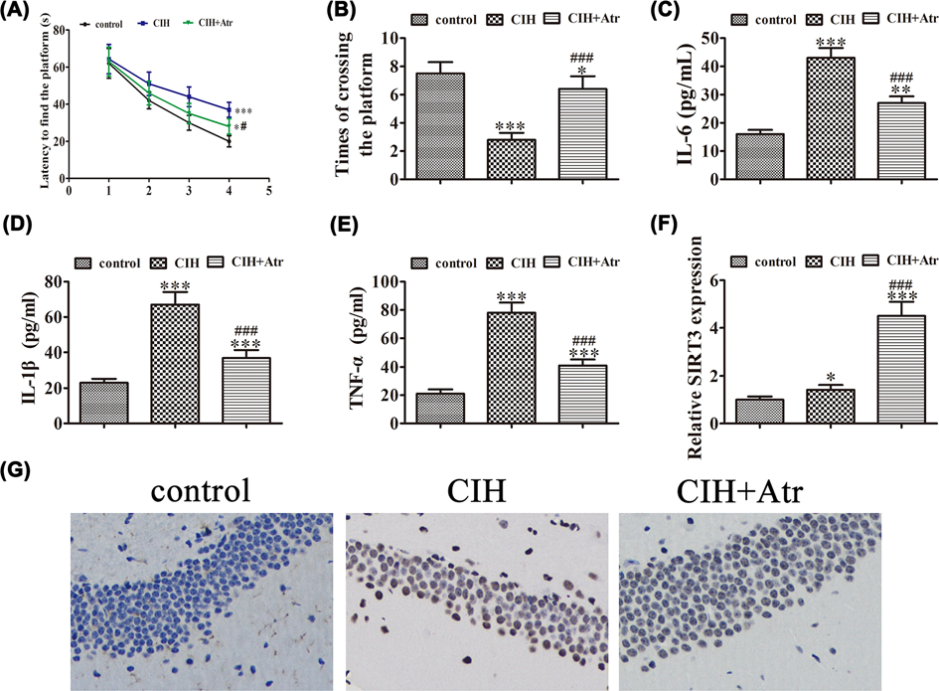
ATR treatment decreased CIH-induced CD and the expression of inflammatory factors in the hippocampal region (**A**) Mice in the CIH group exhibited a significantly longer escape latency than those in the control group. ATR treatment significantly decreased the escape latency. Data are presented as the mean ± standard deviation (SD). *P<0.05, ***P<0.001 versus the control group. (**B**) Platform crossings were decreased in the CIH-treatment groups but increased after treatment with ATR, as compared with the control group. Data are presented as the mean ± SD. *P<0.05, ***P<0.001 versus the control group. ^###^*P*<0.001 versus the CIH treatment group. (**C–E**) The inflammatory cytokines IL-6 (**C**), IL-1β (**D**), and TNF-α (**E**) were measured in the hippocampus by ELISA. Data are presented as the mean ± SD. **P<0.01, ***P<0.001 versus the control group. ^###^*P*<0.001 versus the CIH treatment group. (**F**) qRT-PCR detection of SIRT3 expression in the hippocampal region. Data are presented as the mean ± SD. *P<0.05, ***P<0.001 versus the control group. ^###^*P*<0.001 versus the CIH treatment group. (**G**) TUNEL staining showing injury to nerve cells in the hippocampal region.

To investigate the effect of CIH to the cognitive ability of mice, the MWM test was performed, which is a widely used technique for assessment of spatial learning and memory. The results showed that CIH treatment increased escape latencies when compared with the control group (Figure 1A), whereas ATR treatment significantly decreased the percentage of time in the target quadrant spent by the mice. The results of the spatial probe test showed that CIH treatment decreased the number of platform crossings, while ATR treatment increased the number of platform crossings, as compared with the control group (Figure 1B). These findings indicate that ATR treatment decreased CIH-induced cognitive impairment. ELISA detection showed that CIH treatment promoted expression of IL-6, IL-1β, and TNF-α in the hippocampus when compared with the control group, while ATR treatment decreased CIH-induced expression of these inflammatory cytokines (Figure 1C–E), suggesting that ATR treatment has an anti-inflammatory effect. Then, qRT-PCR detection showed that the expression of SIRT3 was slightly increased in the CIH treatment group, suggesting that ATR treatment up-regulated SIRT3 expression (Figure 1F). According to the previous reports, SIRT3 activation can alleviate ischemia and reperfusion-induced acute kidney injury [18]. Increasing evidence suggests that SIRT3 has anti-inflammatory effects, thus SIRT3 may be involved in ATR-mediated neuroprotection in the hippocampus after exposure to CIH conditions.

The TUNEL method was used to assess apoptosis. The number of apoptotic cells in the control group was low, but significantly increased in the CIH group. The ATR treatment group exhibited decreased apoptosis, as compared with the CIH group (Figure 1G).

### ATR treatment prevents CIH-induced microglial M1 polarization

**Figure 2:**
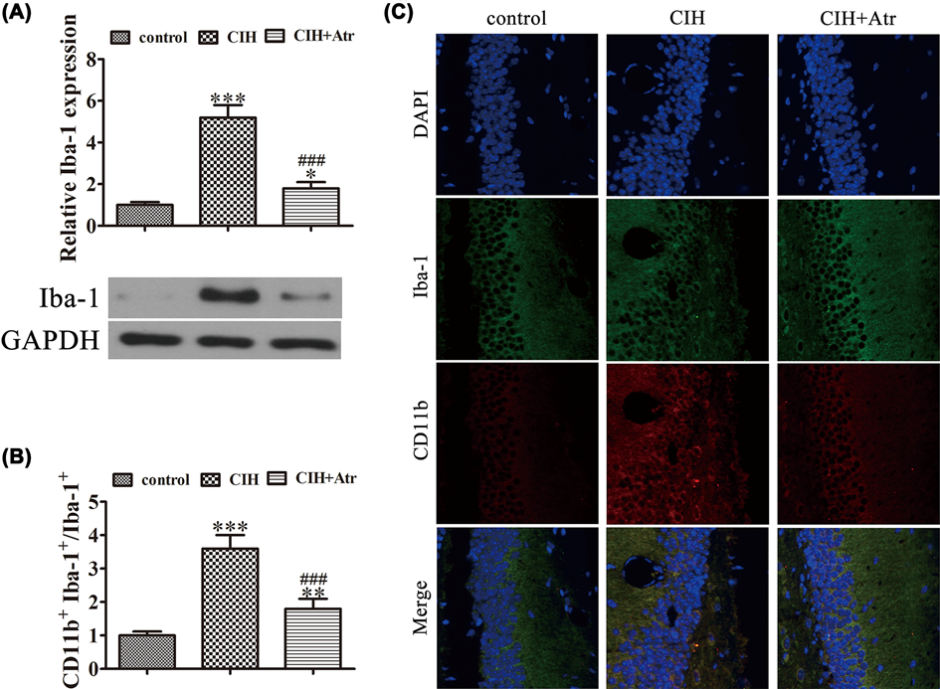
ATR treatment prevented CIH-induced microglial M1 polarization (**A**) Representative protein bands of Iba-1. The relative optical density was normalized to β-actin. Data are presented as the mean ± SD. *P<0.05, ***P<0.001 versus the control group. ^###^*P*<0.001 versus the CIH treatment group. (**B,C**) Immunofluorescence detection of the expression of Iba-1- and CD11b-labeled microglia on hippocampal sections. Data are presented as the mean ± SD. **P<0.01, ***P<0.001 versus the control group.

**Figure 3:**
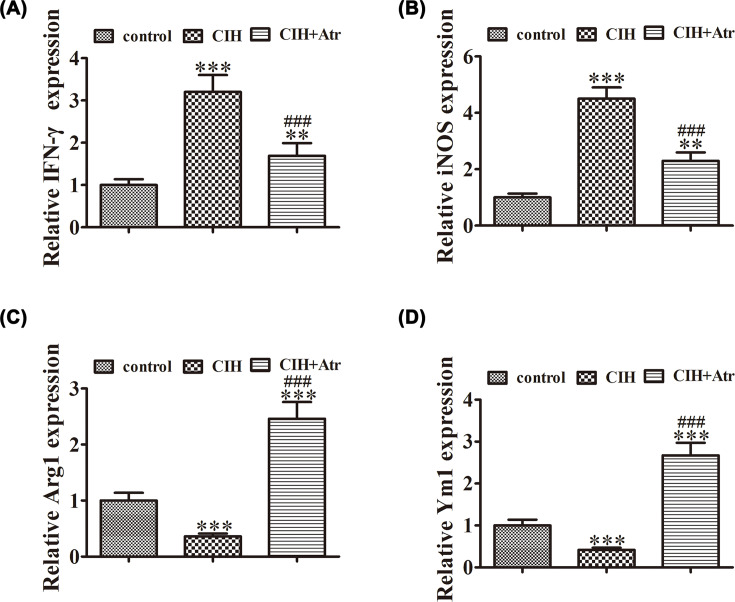
ATR treatment prevented CIH-induced microglial M1 polarization and promoted the M2 microglial phenotype (**A–D**) qRt-PCR detection of the expression of M1 macrophage markers (IFN-γ and iNOS) and M2 macrophage markers (Arg1 and Ym1). Data are presented as the mean ± SD. **P<0.01, ***P<0.001 versus the control group. ^###^*P*<0.001 versus CIH treatment group.

To further determine if ATR conveys a protective effect against the inversion of microglial polarization, western blot analysis was performed. The results demonstrated that Iba-1 expression was up-regulated in the hippocampus after exposure to CIH conditions, suggesting that CIH promoted microglial activation, whereas ATR administration decreased CIH-induced Iba-1 expression (Figure 2A). Immunofluorescent staining showed that CIH promoted microglial activation via up-regulation of Iba-1 expression. The results also showed that CIH promoted CD11b expression in the hippocampus, suggesting that CIH promoted microglial polarization toward the M1 phenotype. In addition, ATR treatment suppressed CIH-induced inflammation by promotion of the M2 microglia/macrophage phenotype (Figure 2B,C). qRT-PCR detection further showed that the expression of M1 macrophage markers IFN-γ and iNOS was increased after exposure to CIH conditions, whereas the expression M2 macrophage markers Arg1 and Ym1 was slightly decreased. But, ATR treatment promoted expression of the M2 macrophage markers Arg1 and Ym1, while suppressing that of the M1 macrophage markers IFN-γ and iNOS (Figure 3A–D). Together, these findings suggest that ATR treatment prevented CIH-induced microglial M1 polarization, while promoting the M2 microglia/macrophage phenotype.

### ATR treatment attenuated CIH-induced damage to BV2 cells by promoting SIRT3 expression and microglial polarization toward the M2 phenotype

**Figure 4:**
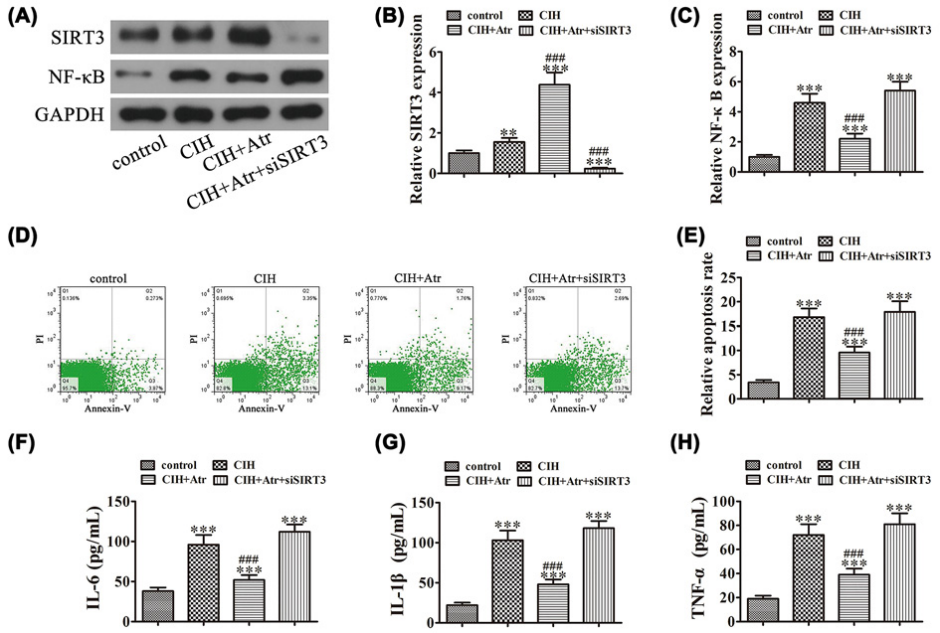
ATR treatment attenuated CIH-induced BV2 cell damage by promoting SIRT3 expression BV2 cells transfected with or without siRNA against SIRT3 were exposed to CIH conditions after pretreatment with ATR. (**A–C**) Western blot showing the expression of SIRT3 and NF-κB in BV2 cells transfected with or without siRNA against SIRT3. Data are presented as the mean ± SD. ***P<0.001, **P<0.01 versus the control group. ^###^*P*<0.001 versus the CIH treatment group. (**D,E**) Cell apoptosis was analyzed by flow cytometry after double-labeling with AV-FITC and PI. The percentage of apoptotic cells in each group was analyzed. Data are presented as the mean ± SD. ***P<0.001 versus the control group. ^###^*P*<0.001 versus the CIH treatment group. (**F–H**) Expression levels of the inflammatory cytokines levels of IL-6 (**F**), IL-1β (**G**), and TNF-α (**H**) in the supernatant were measured by ELISA. Data are presented as the mean ± SD. ***P<0.001 versus the control group. ^###^*P*<0.001 versus the CIH treatment group.

In order to further determine if SIRT3 is involved in CIH-induced microglial injury, BV2 cells were transfected with or without siRNA against SIRT3 to mimic SDB. The study found that SIRT3 expression was slightly up-regulated in response to CIH conditions and ATR treatment significantly promoted SIRT3 expression. Meanwhile, SIRT3 silencing significantly decreased SIRT3 expression. Western blot detection also showed that CIH promoted NF-κB expression. ATR treatment decreased CIH-induced NF-κB expression. After knockdown of SIRT3, NF-κB expression was further increased even with ATR treatment (Figure 4A–C), suggesting that ATR treatment attenuated CIH-induced expression of these inflammatory factors in BV2 cells by the promotion of SIRT3 activation.

Cell apoptosis was analyzed by flow cytometry after double-labeling with AV-FITC and PI. The results showed that ATR treatment reversed CIH-induced apoptosis of BV2 cells, whereas SIRT3 silencing reversed the protective effects of ATR against CIH-induced damage (Figure 4D,E). ELISA detection also found that ATR treatment reversed CIH-induced expression of the inflammatory cytokines IL-6, IL-1β, and TNF-α, whereas SIRT3 silencing reversed the protective effects of ATR against CIH-induced inflammatory responses (Figure 4F–H).

**Figure 5:**
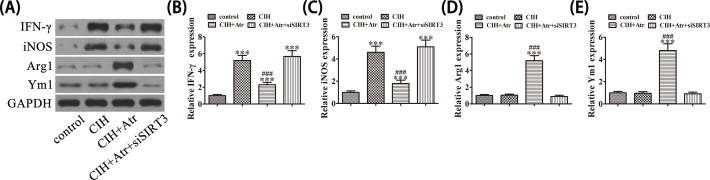
SIRT3 plays an important role in ATR-induced microglial polarization toward the M2 phenotype after expose to CIH conditions (**A–E**) Western blot detection of the effect of SIRT3 on the expression levels of the pro-inflammatory M1 molecules IFN-γ and iNOS, and the M2 molecules Arg1 and Ym1 after exposure to CIH. Data are presented as the mean ± SD. ***P<0.001 versus the control group. ^###^*P*<0.001 versus the CIH treatment group.

Western blot detection showed that ATR treatment reversed CIH-induced expression of the pro-inflammatory M1 molecules IFN-γ and iNOS, while promoting that of the M2 molecules Arg1 and Ym1. Hence, SIRT3 silencing reversed the protective effects of ATR (Figure 5A–E).

## Discussion

The present study showed that CIH can induce CD and up-regulate the expression of inflammatory factors IL-6, IL-1β, and TNF-α in the hippocampal region. Previous studies have found that SDB is a highly prevalent public health concern worldwide that is characterized by repetitive upper airway collapse, leading to intermittent hypoxia, pronounced negative intrathoracic pressure, and recurrent arousal, resulting in sleep fragmentation [11]. Respiratory compromise with SDB is more strongly associated with the systemic inflammation [7]. The accumulation of inflammatory cytokines in the hippocampal region leads to the development of cognitive and memory deficits [17]. We also found that SIRT3 has a regulatory effect in the response to CIH-induced inflammation of the hippocampal region. ATR treatment reversed CIH-induced CD and the expression of inflammatory factors in the hippocampal tissues by promotion of SIRT3 expression.

Sirtuins are identified as nicotinamide adenine dinucleotide (NAD)^+^-dependent class III histone deacetylases. SIRT3 is the only sirtuin that when up-regulated exhibits lifespan-enhancing effects in humans [19]. SIRT3 activity is directly regulated by the NAD^+^/NADH ratio, which is increased under conditions of high energy demand, and up-regulates SIRT3 activity [2]. The results also showed that SIRT3 participates in the regulation of different diseases, especially those associated with the stress response [14]. The results of the *in vitro* experiments showed that SIRT3 silencing reversed the protective effect of ATR against CIH-induced injury to microglial cells and that SIRT3 participated in ATR-mediated regulation of apoptosis of macrophages and microglial cells.

After exposure to stress conditions, such as hypoxia, microglial cells become activated and differentiate into pro- and anti-inflammatory phenotypes. Like macrophages, microglia may exhibit the classical activated M1 phenotype (pro-inflammatory) or, alternatively, the activated M2 phenotype (anti-inflammatory) [1]. CIH induces the differentiation of microglia into the pro-inflammatory M1 phenotype, while ATR treatment suppressed CIH-induced M1 polarization of microglia and promoted differentiation into the M2 microglia/macrophage phenotype. SIRT3 silencing reversed the M2 microglia/macrophage phenotype with ATR pretreatment after exposure to CIH conditions. Together, these results suggest that ATR treatment attenuated CIH-induced damage to BV2 cells by promoting SIRT3 expression and microglial polarization toward the M2 phenotype. These data demonstrate the protective effects of ATR and that ATR treatment prevented SDB-induced CD by suppression of CIH-induced activation of M1 microglia.

## Abbreviations

Ab: antibody
Arg1: arginase-1
ATR: atractylon
AV-FITC: annexin V-fluorescein isothiocyanate
CD: cognitive dysfunction
CIH: chronic intermittent hypoxia
DMEM: Dulbecco's modified Eagle's medium
ELISA: enzyme-linked immunosorbent assay
GAPDH: glyceraldehyde 3-phosphate dehydrogenase
IBA1: ionized calcium-binding adapter molecule 1
IFN-γ: interferon-γ
IL: interleukin
iNOS: inducible nitric oxide synthase
MWM: Morris water maze
NAD: nicotinamide adenine dinucleotide
NF: nuclear factor
PI: propidium iodide
qRT-PCR: quantitative reverse transcription PCR
SDB: sleep-disordered breathing
siRNA: small interfering RNA
SIRT3: sirtuin 3
TNF: tumor necrosis factor
