# Expression of Concern: Atractylon treatment prevents sleep-disordered breathing-induced cognitive dysfunction by suppression of chronic intermittent hypoxia-induced M1 microglial activation

**DOI:** 10.1042/BSR-2019-2800_EOC

**Published:** 2023-05-23

**Keywords:** atractylon, chronic intermittent hypoxia, cognitive disfunctio, microglial, sleep-disorderd breathing

The Editorial Office has been made aware of potential issues surrounding the scientific validity of this paper, hence has issued an expression of concern to notify readers whilst the Editorial Office investigates. It has been noted that the Figure 5A Arg1 blots appear very similar to blots seen in an unrelated manuscript by Xing et al. 2018 (doi: 10.1186/s40659-018-0199-y) and that the control, CIH and CIH+Atr+siSIRT3 bands appearing extremely similar to each other, including identical background features.

